# A Review of State‐of‐the‐Art Microfluidic Technologies for Environmental Applications: Detection and Remediation

**DOI:** 10.1002/gch2.201800060

**Published:** 2018-09-21

**Authors:** Maxine Yew, Yong Ren, Kai Seng Koh, Chenggong Sun, Colin Snape

**Affiliations:** ^1^ Department of Mechanical Materials and Manufacturing Engineering University of Nottingham Ningbo China 199 Taikang East Road 315100 Ningbo China; ^2^ School of Engineering and Physical Sciences Heriot‐Watt University Malaysia No. 1 Jalan Venna P5/2, Precinct 5 62200 Putrajaya Malaysia; ^3^ Faculty of Engineering University of Nottingham The Energy Technologies Building, Jubilee Campus Nottingham NG7 2TU UK

**Keywords:** droplet microfluidics, functional materials, microdevices

## Abstract

Microfluidic systems have advanced beyond natural and life science applications and lab‐on‐a‐chip uses. A growing trend of employing microfluidic technologies for environmental detection has emerged thanks to the precision, time‐effectiveness, and cost‐effectiveness of advanced microfluidic systems. This paper reviews state‐of‐the‐art microfluidic technologies for environmental applications, such as on‐site environmental monitoring and detection. Microdevices are extensively used in collecting environmental samples as a means to facilitate detection and quantification of targeted components with minimal quantities of samples. Likewise, microfluidic‐inspired approaches for separation and treatment of contaminated water and air, such as the removal of heavy metals and waterborne pathogens from wastewater and carbon capture are also investigated.

## 1 Introduction

Environmental pollutions have been trending global concerns following the escalation of anthropogenic activities. While industrialization has been a key driver in economic growth, the trade‐off for environmental conservation has brought about adverse effects as toxic wastes and pollutions are discharged into the ecosystems. Following that, various detection, monitoring, and cleanup technologies, ranging from simple separation techniques to advanced remediation technologies, have emerged. While some of the pollutants can be easily captured and retained following established work on macro‐ or industrial scale, the limit of detections and the costs of current available technologies are still major hitches.^1^ On the other hand, there has been growing amount of publications on the available work of using microfluidic technologies to collect and detect pollutants such as heavy metal ions, volatile organic compounds (VOCs), organic and inorganic ions, fine particulate matters, and even microorganisms in both water and gas samples.^2^, ^3^, ^4^, ^5^

The past decade sees the rapid development of microfluidic technologies in various fields for ubiquitous applications. Miniaturized systems are favorable, especially for analytical purposes, as they require only trace quantities of samples and reagents, effectively cutting down on the time and effort in sampling and sample preparation, with minimal wastes produced. The concept of “miniaturized total analysis system” (μTAS) was coined by Manz et al. whereby some analytical procedures and techniques are integrated to allow all sample handlings at close proximity to where measurements are carried out.^6^ The purpose of μTAS is to enhance the analytical performance of laboratories and tests rather than size reduction of the analytical equipment, yet it has then led to the explosive growth of microfluidic‐based analytical devices.^6^ Analytical procedures that used to be manually handled in laboratories can now be substituted by integrating established techniques such as chromatography, electrophoresis, and flow injection analysis, onto a tiny chip of which the miniaturization is commonly termed as lab‐on‐a‐chip (LOC). Due to the bulk of environmental samples, conventional analytical techniques are often time‐ and cost‐intensive with sample preparation and analyses, resulting in intermittent and slower detections.^2^ Conversely, microfluidics enables the rapid handling of samples. While applications of microfluidic systems are much more common in the areas of biomedical and life sciences, such as DNA and cell assays, researchers have also extended the applications to environmental monitoring and detection where microfluidic sensors are used to detect pollutants such as waterborne pathogens from drinking water, heavy metals and toxic gases from industrial effluents.^3^, ^4^, ^7^, ^8^, ^9^

Many have produced comprehensive reviews on the advances of microfluidic technologies and applications in environmental monitoring and detection. Marle and Greenway first reported on various microfluidic devices for environmental monitoring and demonstrated the different methodologies of detection, while Li and Lin and Saxena et al. further discussed the trends of integrating and miniaturizing the detection systems onto the chips, or coupling of microdevices to larger apparatus, with various multianalyte methods for sample processing and analysis having been reviewed by Jokerst et al.^2^, ^10^, ^11^, ^12^ Both Jang et al. and Giri Nandagopal et al. published all‐inclusive studies on the advanced approaches on assessing and monitoring water, air, and soil pollutants.^13^, ^14^ Notably, there has been explosive growth of applications of existing miniaturized systems for environmental analysis and novel creative techniques that address and resolve fundamental issues concerning sample introduction, size of sample volumes, matrix interferences, and the limits of detections.^10^, ^11^

To the best of our knowledge, no comprehensive review has been performed on microfluidic inspired innovations for environmental remediation, such as the use of microfluidic‐synthesized functional materials in pollutant separation and removal. Droplet‐based microfluidics is a subcategory microfluidics that centers on the generation and manipulation of discrete droplets with microdevices. It has recently emerged as a promising platform for designing and production of functional materials that can be used for environmental remediation.^15^, ^16^ The advancement of droplet‐based microfluidics offers mature drop generation and manipulation techniques which allows controlled production of microemulsions and capsules with core–shell structure.^17^, ^18^ This review aims to provide an all‐inclusive review on microfluidic systems for environmental applications–monitoring and detection of contaminants, as well as removal of pollutants.

## 2 Microdevices for Monitoring and Detection of Pollutants

Microdevices possessing fluidic channels and miniaturized analytical equipment are emerging as portable devices ideal for on‐site sampling. The development of handheld electrochemical sensors and biosensors for pollutant sampling has partly resolved challenges faced by laboratory‐based analyses, such as lengthy preparation time and quality change in samples prior to analysis, while LOC sensors assimilated various conventional procedures into a single system.^19^ Some of the advantages of miniaturized features in microfluidic systems include rapid analysis, low sample and reagent demand, and real‐time characterization.^11^, ^20^ A portable microdevice may also contain an on‐chip pretreatment section, channels in which samples flow, and a miniaturized detector which may be connected to an analytical unit. Recent researches have shown trends of miniaturizing analytical units such as portable and fieldable mass spectrometry and micromachined fluorescence detectors with microlenses, allowing instantaneous detection without the need of a full‐fledged laboratory.^21^, ^22^

### 2.1 Sample Preparation and Preconcentration

Prior to any chemical or analytical processes, bulk water or air samples have to be preconcentrated before separation due to the low concentrations of pollutants in samples typically in the range of parts per million (ppm) or parts per billion (ppb). Preconcentration of environmental samples in microdevices can be achieved through the integration of an automated pretreatment module onto the device, which eliminates any arduous and labor‐intensive operations and prevents sample handling loss or contamination.^11^

On‐chip preconcentration technologies are generally classified as static and dynamic mechanism–based techniques.^23^ The former refers to extraction and isolation of contaminants with the aid of a functionalized solid support, such as solid‐phase extraction, absorption, or filtration; while the latter by manipulating the electrokinetic properties of pollutants to achieve selective enrichment, via electrophoretic stacking, focusing, and sweeping.^4^, ^12^, ^24^, ^25^ An integration of solid phase extraction (SPE) and capillary electrochromatography (CEC) had been demonstrated on an on‐chip packed‐bed chromatography. Oleschuk et al. reasoned that the utilization of packed beds in microsystems for sample concentration had not been greatly explored due to the complexity of loading the beads or stationary phases.^26^ The group successfully loaded octadecylsilane (ODS)‐coated beads into a 330 pL chromatographic bed of 200 µm long on a glass substrate via electrokinetic pumping. 1.0 × 10^−9^ m of nonpolar analyte BODIPY 493/503, a fluorescent dye was retained on the bed and then eluted with the CEC.

At low Reynolds number <10^2^, conventional liquid–liquid laminar extraction in microdevices is ineffective, due to slow interfacial exchange rate within short path lengths.^27^ To promote convective flow and mixing, partition walls and different structures have been suggested to generate slight perturbation and promote convective mixing via secondary flow.^28^ For instance, a cross‐shaped structure incorporated into liquid–liquid extraction microdevice improved the extraction efficiency by 350% compared to traditional laminar flow extraction.^29^ Analyte could also be extracted from the bulk sample through a thin membrane via the liquid phase microextraction (μLPME) device.^30^ A polypropylene membrane impregnated with 1 µL of dihexyl ether was sandwiched between two poly(methyl methacrylate) (PMMA) plates. The device was used to primarily detect the presence of pharmaceutical wastes in the environment, and it had also been tested on nonsteroidal anti‐inflammatory drugs in biological and environmental samples, with high extraction efficiencies of over 72%. Despite the reduced extraction time of only a few minutes, the drawback of this technique is that it supports only stopped flow analysis, after which the sample would then have to be removed and tested with the high performance liquid chromatography (HPLC).

Capillary electrophoresis (CE) is widely used in the separation and transport of various types of analytes, and has also been used in concentrating pathogens and microorganisms.^31^ Balasubramanian et al. developed a microdevice for continuous capture and concentration of microorganisms and tested on concentrating various bacteria (*Escherichia coli*, *Salmonella*, *and Pseudomonas*) and viruses (*Enterobacteria phage MS2* and *Echovirus*) in reclaimed and bottled water samples.^32^ The capture efficiency was found to differ for each type of species and to be a function of time, flow rate, as well as voltage applied. Generally, the device showed remarkably high capture efficiency without the need for any chemical additives. Likewise Puchberger‐Enengl and Vellekoop utilized a glass chip with hydroxyapatite (HA)‐doped organic/inorganic sol–gel (Ormosil) on platinum electrodes to study the adhesion of *Saccharomyces cerevisiae* cells.^33^ Continuous separation was achievable with flow at 14 µL min^−1^ with a concentration factor of 27.7 ± 3.4 within 20 min.

**Figure 1:**
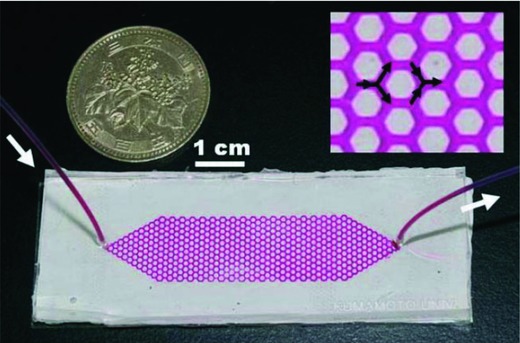
Honeycomb‐structured microchannel scrubber. Colored solution is introduced to show the channel, and the inset is a close‐up of the channel. Reproduced with permission.^4^ Copyright 2005, Royal Society of Chemisty.

Gas preconcentration and analysis, on the other hand, are even more challenging as there are possibilities of gas leakage and adsorption onto other substrates during transport. Toda et al. developed a microchannel scrubber made of poly(dimethylsiloxane) (PDMS) with a PDMS gas permeable membrane of 7 µm thickness for concentrating hydrogen sulfide in the inflow gas.^25^ Hydrogen sulfide came into contact with a fluorant‐containing scrubbing reagent later conveyed for fluorescence detection. A honeycomb‐structured microchannel such as that shown in **Figure** **1** was subsequently proposed for the simultaneous measurements of H_2_S and sulfur dioxide, SO_2_.^4^ A porous polytetrafluoroethylene (pPTFE) membrane of 30 µm thickness was attached onto the microchannel PDMS plate for the collection and permeation of targeted species. A honeycomb structure is advantageous in that it has a larger absorbing area, and should there be any defect or clogging, liquid would be able to bypass and flow into another channel. For on‐site gas analysis, a gas concentration system was developed to calibrate micro‐gas analysis system for more reliable measurements of SO_2_, H_2_S, and CH_3_SH. The honeycomb‐structured microchannel was used as a micro‐gas desorber to strip and concentrate gases generated from the source reagent solutions before transferring the gases into another microchannel scrubber with fluorant‐containing scrubbing agent for fluorescence detection.^34^ Li et al. on the other hand created a microfluidic gas centrifuge that allowed a twofold enrichment of diluent based on the molecular weights of sample constituents within 0.01 ms.^35^

Microparticles and microorganisms can also be sorted through the simple use of geometries such as spiral channels.^36^, ^37^ Cryptosporidium cells with an average size around 4–5 µm that can go undetected in water bodies were separated from wastewater via inertial focusing in multiple loops spiral channels.^37^ The spiral channel had an inlet in the middle that spiraled out to two outlets for the concentrating of particles and purging of remaining liquid. The separation of pathogens occurred with the balancing of the net lift forces and the drag induced by the curved channel, as the pathogens settled into an equilibrium position without propagation of any external force. The focusing behavior of the pathogens was subjected to the shape and size of the targeted species and could be controlled by manipulating the applied flow rate and the concentration of the particles.

The techniques for concentrating analytes of large‐volume samples are rather well‐developed, yet in doing so for ultrasmall volume samples, challenges lie in enhancing the selectivity and sensitivity of microdevices especially within confined spaces in tiny reservoirs and microchannels with limited heat and mass convection.^31^ The integrated processes of sample pretreatment, separation, and detection significantly cut down on the range of sample preparation steps and reduce problems with on‐chip detection limits.^26^ The selection of detection methods and related coupling to the microdevice depends on the required limit of detection, selectivity, and sensitivity, and the anticipated multifunctionality and the integration and interfacing with other processes and instrumentations.^11^, ^38^ Monitoring and detections on lab‐on‐a‐chip systems are broadly categorized into three major methods: optical detection, electrochemical detection, and mass spectrometry, with optical and electrochemical detection methods more commonly utilized owing to the ease of use, flexibility of being scaled down, as well as the wider range of applications available.^10^, ^12^, ^39^

### 2.2 Optical Detection

Optical detection involves the monitoring and detection of light properties such as absorbance, fluorescence, and luminescence patterns emitted from the samples upon excitation. Nitrite levels in drinking water and water bodies are constantly monitored as excess will cause adverse effect to both human and marine life.^40^ Nitrite level in water can be determined by analyzing the UV absorption of nitrite samples.^41^ And, using colorimetric methods with Griess reaction, Sieben et al. extended the absorbance path length of a nitrite detection system to 25 mm to detect nitrite at detection range of 50 × 10^−9^ m to 10 × 10^−6^ m, with a limit of detection of 14 × 10^−9^ m.^40^ Fujii et al. demonstrated the dual detection of sulfite and nitrite in an aqueous solution fluorometrically with fluorescent agents *N*‐(9‐acridynyl)maleimide (NAM) and 2,3‐diaminonaphthalene (DAN).^42^ Sulfite and nitrite are from post‐combustion exhausts and the latter is also found in runoff of fertilizers. As both the respective fluorescent reagents for sulfite and nitrite, NAM and DAN have relatively close wavelengths of excitation which were picked up by a commercial fluorescence spectrophotometer. Takabayashi et al. also used DAN for the detection of atmospheric nitrogen dioxide in a quartz glass microchip.  NO_2_ in the atmosphere flowed through a porous glass plate embedded in the chip into triethanolamine (TEA) solution flowing within the channel, producing NO_2_^−^ ions which could be detected under the excitation of ultraviolet light‐emitting‐diode (UV‐LED).^43^ The microdevice was able to detect and quantify NO_2_^−^ ions below 1 fmol, corresponding to atmospheric nitrogen dioxide in the range of 10–80 ppbV.^43^ Toda et al. who experimented on hydrophobic polymer as gas permeation layer, however pointed out that porous glass plates might cause the absorbing solution to seep into the pores, likewise the gas might get caught in the pores as well.^25^

In gas–liquid systems, challenge may arise from the formation of gas bubbles when both the phases are mixed directly. Gao et al. replaced standard use of porous glass or permeable membrane interface with a thin layer of luminol solution held between two convex structures by surface tension alone.^5^ The basic luminol solution was used as an absorption reagent of chlorine gas in chemiluminescence detection in glass microdevice. This method yielded detection limit of 0.2 ppm for standard chlorine gas. The remarkable advantage of the detection system lies in that both adsorption and detection were carried out at the gas–liquid interface and hence the appearance of bubbles could be avoided. While conventional microdevices are mostly fabricated of glass or PDMS, paper‐based sensors or paper‐based analytical devices (PADs) are functionable without an external fluid driving pump, and have gained wide popularity due to its simplicity in fabrication and benefit of fast analyses.^44^ Also when combined with other available detection methods, the increased sensitivity of PADs makes it possible to be used for quantitative analyses with almost always instantaneous results/measurements.^38^ A miniaturized chemiluminescence detection system for Cr(III) ions on a microfluidic PAD had been developed and the micro PADs *(μ*PADs) were used to determine Cr(III) level in tap water and other water bodies.^45^ The results obtained were comparative to that obtained by inductively coupled plasma optical emission spectroscopy (ICP‐OES). Under the optimal conditions, a linear range was obtained from 0.05 to 1.00 ppm with a detection limit of 0.02 ppm.

The use of Raman‐microscopic systems has received increasing attention in recent years. As a nondestructive approach for analysis of gaseous, aqueous, and solid samples, Raman spectroscopy can facilitate superior analysis at microfluidic scales as it can enable high spatial resolutions.^46^ Real‐time detection of dipicolinic acid (DPA) and malachite green (MG) has been successfully demonstrated by Quang et al. with a portable Raman spectrometer coupled with a micropillar array chip.^47^ Both the substances are adsorbed onto silver nanoparticles in a PDMS microchannel whereby mixing is promoted by the pillar array–induced convective flow. The combination of droplet‐based microfluidics with surface‐enhanced Raman scattering (SERS) was also reported for trace analysis of mercury(II) ions in water. Rhodamine B (RhB) molecules originally adsorbed onto gold nanoparticles (AuNPs), RhB‐adsorbed AuNPs, were released in the presence of mercury(II) ions. This was due to the stronger affinity between mercury(II) ions and AuNPs, and the concentration of mercury(II) ions was expressed in terms of the changes in the SERS signals.^48^

### 2.3 Electrochemical Detection

When subjected to an electrical field, electroactive species or electrolytes with ionic species can be efficiently separated and detected. A micellar electrokinetic chromatography with electrochemical detection (MEKC‐EC) was proposed for the separation and detection of trace phenolic compounds in water samples amperometrically.^49^ The samples were first preconcentrated with field‐amplified sample stacking (FASS) and field‐amplified sample injection (FASI) approaches. A screen‐printed carbon electrode modified with cellulose–double‐stranded DNA was used to amplify the sensitivity during the electrooxidation of the eight phenolic compounds. Nie et al. proposed another form of modified carbon disk electrode with mesoporous carbon material (CMK‐3) for the simultaneous detection of four types of important nitroaromatic compounds (NACs), including 2,4,6‐trinitrotoluene (TNT), 1,3,5‐trinitrobenzene (TNB), 2,4‐dinitrotoluene (DNT), and 1,3‐dinitrobenzene (DNB) in several water samples such as tap and river water and coking wastewater.^50^ These NACs are known to be highly toxic and carcinogenic. The coating CMK‐3 has high electrical conductivity and improved sensitivity which is beneficial for the detection of trace NACs of 3.0–4.7 µL g^−1^, while common electrodes can hardly give any higher sensitivity.

**Figure 2:**
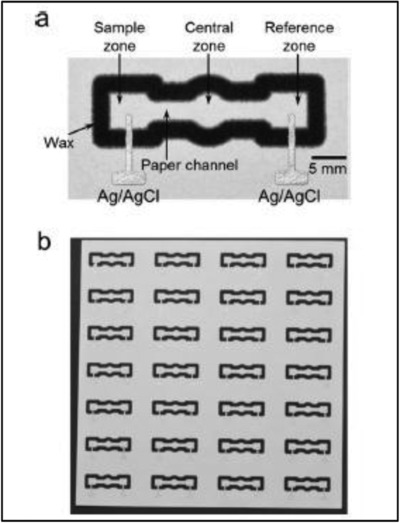
a) Photography of a Cl^−^ sensing EPAD, with defined zones. b) 28 potentiometric Cl^−^ sensing EPADs fabricated on one page of wax‐printed paper (20 × 20 cm^2^). Reproduced with permission.^52^ Copyright 2014, American Chemical Society.

Ion‐selective electrodes (ISEs) are vastly used in potentiometric detection to continuously measure ion activity in untreated samples.^51^ Determination of Cd^2+^, Cl^−^, Pb^2+^ ions and the pH of solutions had been carried out by holding an ISE and a reference electrode vertically and pressing lightly onto a paper soaked in liquid sample.^51^ The use of papers for sampling is advantageous where the samples have high content of solids preventing effective measurements, and it may also protect the active surface of potentiometric sensors from contamination and fouling by solid impurities. Another ion‐sensing electrochemical paper‐based analytical device (EPAD) was proposed for measuring concentrations of electrolyte ions (Cl^−^, K^+^, Na^+^, and Ca^2+^), and the reference electrode of Ag/AgCl was stencil‐printed onto the wax‐printed chromatography paper, as shown in **Figure** **2**.^52^ The geometry of the EPAD allowed the slow diffusive transport of ions across the paper channels when both sample and reference solutions are introduced. The drawback of EPADs at current stage, however, is the lack of stability in electromotive force (EMF) measurement that can result in more than 10% error relative to conventional measurements. Nevertheless, EPADs are still very promising in that they are easy to fabricate without any complex techniques and are readily adaptable to any portable electrochemical readers for the measurement of potential differences.

Conductivity detection (CD) can be contact or contactless. In the contact mode, the electrodes in galvanic contact with the working solution is exceptionally effectual for the analysis of small ions.^12^ Contactless detection can be used with a wide range of background electrolytes and point of detection is flexible. An on‐column direct current (DC) conductivity detection system for detecting potassium ion was fabricated on a glass chip with double‐T‐shaped structure.^53^ Platinum wire was used to generate electrophoretic current throughout the separation zone. As the two sensing electrodes were separated from direct contact with the sample, the issue of electrodes polarization was avoided. This method achieved a detection limit of 15 × 10^−6^ m (600 ppb or 900 fg) of potassium ion in a 2 × 10^−3^ m Tris‐HCl buffer (pH 8.7) with a linear range of 2 orders of magnitude without any stacking. A contactless conductivity detector was integrated into a PDMS microchip which completely insulated the electrodes from the measuring solution.^54^ The chip successfully separated and detected several inorganic ions and also a mixture of heavy metal ions with lowest detection limit at 0.4 × 10^−6^ m. Different detection techniques have been coupled for the simultaneous detection of different species and for improved performances.

### 2.4 Mass Spectrometry and Other Detection Techniques

Mass spectrometry (MS) performs separation and detection based on their mass‐to‐charge ratio, and is most regularly coupled with biological assays for laboratory analysis needing high sensitivity and low limit of detection. In the past, due to the bulk of MS, as well as the advanced sample preparations needed, this technique was not highly sought after especially for quick and on‐site analyses. To date, several coupling methods of microdevices to off‐chip mass spectrometers have already been developed, and the miniaturization of mass spectrometers to fieldable sizes has also been reported. Snyder et al. summarized the advances in miniaturization of mass spectrometers of which individual components such as sample ionization, ion transportation, analyzers, and even the miniaturization of power and data systems to suit the needs of on‐site field analyses.^21^ One of the challenges for coupling microdevices to MS, however, is the reduced flow rate of samples from the device, and Wang et al. detailed different methods of coupling to current available ionization methods via analog or channel‐based, digital, and droplet microfluidics.^55^ An ion trap–based palm portable MS weighing 1.48 kg and working on batteries had been successfully developed for direct detection of toluene and dimethyl methylphosphonate (DMPP) in air at detection limit of 6 and 22 ppm, respectively.^56^ Other researchers such as the groups of Ramos‐Payan et al. and Iwata et al. demonstrated innovative designs of liquid‐phase microextraction of double‐flow microdevice and smart gas sensing system of microhotplates, respectively.^57^, ^58^ **Table** **1** summarizes the current available detection methods with microfluidic systems.

**Table 1:** A summary of detection methods with microfluidic approach (note: m denotes molar)

Detection method	Core technological component	Class of pollutants	Detection limit	Reference
Optical	Laser‐induced fluorescence	Sulfite and nitrite in aqueous solution	1 × 10^−6^, 0.4 × 10^−6^ m	42
	UV‐LED excitation, fluorescence detection	NO_2_	30–200 ppbV	43
	On‐chip microfluorescence detector	H_2_S	1 ppbV	25
	Absorbance detection with optofluidic modulator	Methylene blue	7 × 10^−3^ m	59
	Griess method for nitrite detection on chip	Nitrite in drinking water	14 × 10^−9^ m	40
	Miniaturized chemiluminescence detection on paper‐based device	Chromium(III)	0.02 ppm	45
	Gas–liquid chemiluminescence of luminol–chlorine system	Chlorine gas	0.2 ppm	5
Electrochemical	MEKC‐EC	Trace phenolic compounds	100 × 10^−12^–150 × 10^−12^ m	49
	Capillary electrophoresis with amperometric detection	Nitroaromatic compounds	3.0–4.7 µg L^−1^	50
	Paper‐based ion‐selective electrode (ISE)	Cd^2+^, Cl^−^, Pb^2+^	1 × 10^−6^–0.1 × 10^−3^ m	51
	Paper electrodes and ion‐selective membrane	K^+^, Na^+^, Cl^−^, Ca^2+^	1 × 10^−3^–146 × 10^−3^ m	52
	On‐column direct current conductivity detection	K^+^	15 × 10^−6^ m	53
	Capillary electrophoresis with contactless conductivity detection	Heavy metal ions	0.4 × 10^−6^ m	54
MS	Pulsed gas sampling in ion trap assembly on palm portable mass spectrometer	Toluene and dimethyl methylphosphonate (DMMP)	6.4, 52.9 ppm, respectively	56
SERS	SERS with droplet‐based microfluidics	Mercury ions	100–500 ppt	51
	SERS with micropillar array microchannel	Dipicolinic acid and malachite green	200, 500 ppb	50

## 3 Microfluidics for Environmental Remediation

The environment is on the line due to excessive discharge of harmful by‐products from industrial activities in the face of growing economies. As the demand for clean resources grows with urbanization and industrialization, it becomes exceedingly challenging to effectively handle the voluminous waste emitted either into water bodies or into the atmosphere. As conventional cleanup technologies are becoming inadequate in meeting effluent limits regulatory, more efficient and cost‐effective alternatives are highly sought after. The selection of each treatment technique, either in treating water or air discharge, is subjected to various factors, such as the concentration and types of waste, required level of cleanup, and choice of postprocessing technologies.^60^ While every existing treatment technology has its own strengths and shortcomings, this review is primarily focusing on recognizing published work in microfluidics as potential alternatives for environmental remediation. Indubitably, scaling up remains the biggest challenge in adopting microfluidic technologies, yet the use of microfluidics has shown very promising results and has also enabled more studies to be done.^61^

### 3.1 Microfluidic Reactors

**Figure 3:**
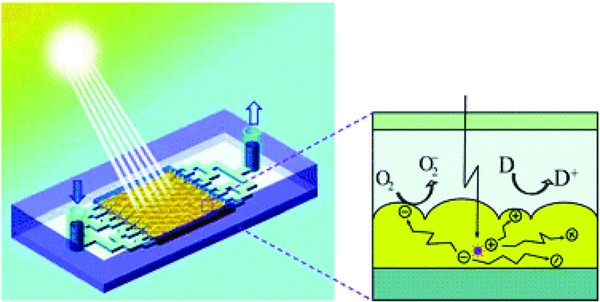
An illustration of a photocatalytic microreactor whereby oxidation and reduction reactions are induced by photoexcited electrons to decompose water contaminants. Reproduced with permission.^63^ Copyright 2014, Royal Society of Chemistry.

Photocatalytic water treatment has been a difficult yet trending research topic for the past two decades, drawing in interests across multiple science disciplines.^62^ The photocatalytic process involves the decomposition of organic pollutants into harmless products, such as carbon dioxide and water, using visible light.^63^ Various configurations of microfluidic reactors have been reported and used in photocatalytic water treatment, especially in treating organic dyes in wastewater. While the term microfluidic reactors may suggest any reactions in microscale reactors, this section provides the prospect of using miniaturized reactors for water purification, such as that illustrated in **Figure** **3**; and should not be confused with droplet‐based microreactors where reactions occur within droplets. The advantages of using microreactors for water purification include much shorter reaction time and uniform irradiation over significantly extended reaction surface area. Thin films and nanomaterials have been described as excellent photocatalysts, with zinc oxide reported as an interesting alternative to titanium dioxide (TiO_2_) which is seen as the ideal material for photocatalysis.^64^, ^65^ A microreactor containing zinc oxide nanowires as photocatalytic medium had successfully degraded five volatile organic compounds: benzene, toluene, ethylbenzene, *meta*‐, *para*‐, and *ortho*‐xylenes (BTEX) in water to the corresponding permissible level established by the US Environmental Protection Agency.^65^ Despite the advances in materials development and various seemingly promising configurations such as integrated photocatalysis–thermolysis water purification, low throughput in microreactors is still a great impediment to its usage.

### 3.2 Environmental Treatment with Droplet‐Based Microfluidics

**Figure 4:**
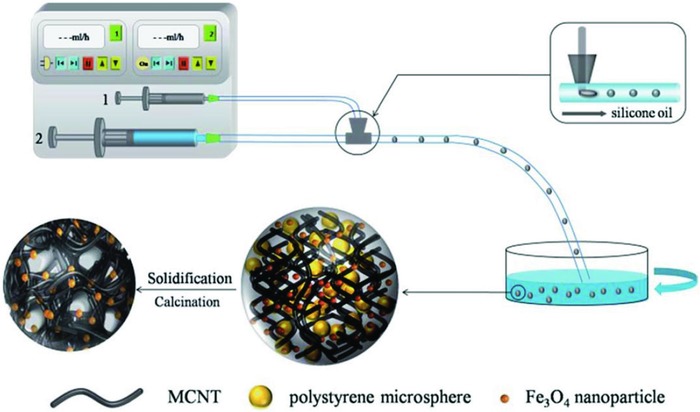
Droplet‐based microfluidics for producing functional microparticles in microdevices. MCNTBs were produced in a modified T‐junction device. Reproduced with permission.^78^ Copyright 2016, Royal Society of Chemistry.

Droplet‐based microfluidics has recently received great attention in fabricating emulsions of uniform sizes.^66^ Despite there being two general methods of producing emulsions, active and passive, this section mainly focuses on the passive formation of emulsions, which represents the breakup of discrete phase in continuous phase driven by hydrodynamic pressure and flow instabilities without external actuations.^15^ Emulsions can be generated and manipulated in microdevices of different geometries such as that shown in **Figure** **4**, yielding different sizes and morphologies. Extensive studies have been conducted on droplet microfluidic device fabrication, device geometries, and breakup mechanisms.^17^, ^67^, ^68^, ^69^ Conventional methods of producing droplets such as emulsion polymerization, suspension polymerization, or evaporation‐induced consolidation, usually do not guarantee high monodispersity.^70^ Microfluidic approach allows precise control over size, shape, and morphologies of microdroplets. Arguably, the production of monodisperse emulsions is on a drop‐by‐drop basis which may result in low throughput, yet production rate may be increased through parallelization of devices and extension of microchannel networks.^71^

Emulsions are used as templates for the production of functional microparticles, such as Janus microparticles, core–shell microemulsions, and hybrid microparticles which have been developed with the advances of microtechnologies.^72^, ^73^ By regulating channel geometries and flow rates, emulsions can be compartmentalized into containing several different domains and can be used as microcarriers of multiple ingredients. For multiple emulsions, a shell layer usually encapsulates the content within and provides a certain form of protection, whether to or from the environment. A ubiquitous application is core–shell‐structured capsules for drug release, which when triggered by different stimuli, release the active ingredients within.^18^ Other known functions of the external solid shells are semipermeable membranes that selectively allow the passing of explicit particles; and stimuli‐responsive membranes that only release the encapsulant corresponding to different stimuli, such as temperature, pH, and concentration of specific substances. Other configurations of multiple emulsions are also possible with good controllability over each components with microfluidic approach. Metal nanoparticles such as Fe_3_O_4_ have also been incorporated into microparticles for functionalization purposes.^74^

There have been numerous reports on the extensive development of functional polymeric microparticles with advanced functions from controllable microfluidic emulsions for drug delivery and controlled release, and other applications such as cell encapsulation and synthetic biology.^70^, ^75^, ^76^ In the following sections, applications using functional microparticles generated via droplet microfluidics for removal of toxic pollutants will be presented.

#### 3.2.1 Water Decontamination

Adsorption has always been an important and established technique in waste treatment, regardless of handling liquid or gas phase. With growing interest in sorbent studies, more low‐cost sorbents and alternative materials that possess enhanced adsorption capacities, such as polymeric matrices have been developed.^77^ Microfluidic approaches are great alternatives for adsorbent preparation as they allow the control of the space structure of the emulsions.^78^ Microparticles also make good sorbents as they have higher surface to volume area and their characteristics such as affinity, selectivity, and porosity can be easily modified.

Biosorbents are especially favorable, such as activated carbon and chitosan. Chitosan is a promising biosorbent due to its abundance and performance, and has been known as being an effective sorbent for removing heavy metals from coal mining wastewater, and has been used as the packing sorbent materials in column adsorber for the removal of oil from the wastewater stream produced in oil industry.^79^, ^80^, ^81^ The performance of chitosan sorbents can often be significantly improved via purpose‐oriented surface modifications and some of the modified chitosan adsorbents include chitosan‐bound Fe_3_O_4_ nanoparticles, chitosan–cellulose hydrogel beads, silica/chitosan composite, magnetic Cu(II) ion–imprinted composite.^82^

Chitosan microspheres have been conventionally prepared via emulsion polymerization or emulsification–diffusion method.^83^ A PMMA microchip having a coaxial structure, with a stainless steel needle as the dispersing channel, had been used to synthesize hybrid chitosan–silica microspheres microfluidically.^84^ Silica was added to the microspheres to enhance the mechanical properties and the removal of copper ions in water. Pores were induced in the microspheres with the addition of polyethylene glycol (PEG) which is a foaming agent, resulting in an increased specific surface area, hence improved adsorption rate. The dispersed emulsions were transferred and solidified in a mixture of glutaraldehyde and *n*‐octane with the former as the cross‐linking reagent. The microemulsions showed good sphericity and monodispersity with a sponge‐like structure. The hybrid chitosan microspheres with spongy pores reached adsorption equilibrium faster at faster adsorption kinetics than the nonhybrid ones and had greater adsorption capacity at 53.0 mg g^−1^ than the latter at 43.0 mg g^−1^ adsorbent.

Zhu et al. also reported the synthesis of chitosan microspheres with a PMMA microfluidic chip with cross‐junction and orifices.^82^ The chitosan microspheres had been modified via graft modification using thiourea to increase the presence of amino functional group such that the removal of copper ions in waste water could be enhanced. Porous structure was induced with the use of PEG, at an optimum ratio of 2:1 to chitosan mass; similarly cross‐linking of microspheres were carried out in a solidification bath containing glutaraldehyde with continuous stirring. The microspheres retained their porous structure and were later treated with thiourea containing acetone solution in a thermostat water bath. Through linear titration method, amino content in both nonmodified chitosan microsphere (NMCM) and thiourea‐modified chitosan microsphere (TMCM) was found to be 6.88% and 14.08%, respectively, with a significant increase in the latter upon graft modification. Most copper ions were removed via chemical adsorption as the copper ions were chelated or form coordination bonding with the electrons transferred from nitrogen atoms. The performance of TMCM was compared with that of NMCM, whereby TMCM exhibited higher adsorption capacity of 60.6 mg g^−1^ of Cu(II) ions. Zhu et al. also tested the microsphere adsorption capacity for Na(I), Al(III) in different combinations at various concentrations, however the microspheres appeared to show greater affinity for Cu(II).

Carbon nanotubes (CNTs) have been widely used in water treatment, of which some of the uses include adsorptive media for various organic and inorganic pollutants,^85^ and catalysts for degradation of water pollutants.^86^ Its lightweight, high total surface area and porosity, and recyclability are promising traits of CNTs; nonetheless, low density and poor wettability are the main setbacks for the development of CNTs into efficient adsorbents. In an attempt to remove oil and various organic solvents such as toluene and chloroform from contaminated water, Cao et al. used a modified T‐junction microdevice producing porous beads containing multiwalled carbon nanotube.^78^ The multiwalled carbon nanotube beads (MCNTBs) exhibited effective adsorption capacities of up to 6–18 times their own weight. Polystyrene microspheres (293 ± 10 nm) were added to a mixture of acidified MCNTs and Fe_3_O_4_ nanoparticles and later calcinated to induce porous structure; while the Fe_3_O_4_ nanoparticles gave the beads the magnetic properties for ease of recovery and motion control. The MCNTBs had uniform diameters of ≈200 ± 10 µm, and possessed superhydrophobic and oleophilic properties which are vital in adsorbing oil and organic pollutants. Graphene was later incorporated into the MCNTBs and magnetic porous graphene/multiwalled carbon nanotube beads (MPGCBs) were obtained by the same group via the same fabrication method.^87^ The MPGCBs had been tested on the contaminated water containing pollutants that had been previously carried out on the MCNTBs, and a significant increase in the adsorption capacities was observed. The MPGCBs effectively removed pollutants of 8–25 times its own weight, which was higher than the absorption capacities for conventional sorbents such as activated carbon, zeolite, saw dust, etc. Both MCNTBs and MPGCBs were recovered and the recoverable substances could be distilled, with at least 6 adsorption–distillation cycles having been carried out, while nonrecoverable pollutants were combusted. Copic et al. produced CNT supraparticles of 97.2 ± 10.7 µm and packed them into microfluidic column filters to remove sodium dodecyl sulfate (SDS), an anionic surfactant found in wastewaters.^88^

#### 3.2.2 Carbon Capture and Storage

Carbon capture and storage (CCS) has widely been regarded as an indispensable pathway in containing atmospheric CO_2_ without compromising energy security. Conventional techniques for large‐scale carbon reduction include chemical and physical absorption, adsorption with solid sorbents, selective separation with membrane systems, and also cryogenic separation.^89^ Amine scrubbing is deemed the most mature technology for carbon removal from processed gases yet the major drawback is its prohibitive energy penalty. Many other alternative capture technologies are still under intensive development and the majority of them are yet to be fully demonstrated and commercialized. Among the many capture technologies currently under development, adsorption‐based carbon capture has been recognized as being a viable alternative, both technically or economically, and various types of solid sorbents have been investigated, including supported amines, supported carbonates, zeolites, and different classes of microporous organic polymers (MOPs).^89^, ^90^ MOPs have shown good thermal and chemical stability, better performances, and selectivity toward various gases, and are promising in that they show great potential in synthetic diversification.

Kaliva et al. first reported on the facile use of microporous polystyrene particle for biogas purification.^91^ Microporous poly‐styrene particles were produced from an emulsion free‐radical copolymerization process with divinylbenzene (DVB). The particles of average diameter of 40 nm formed agglomerates and had ultra‐micropores of ≈4–6 Å. The microporous polystyrene particles provided a larger surface area and exhibited excellent separation of polar gases such as CO_2_ in biogas purification, even in the presence of moisture. Other reported works on producing MOPs for CO_2_ capture include decorating nanocavities in mesoporous polymer particles with tunable amine for CO_2_ molecule recognition properties.^92^ Most of these microporous spherical adsorbents are still fabricated via conventional methods of seeded polymerization, emulsion polymerization, whereby the size of the microparticles is determined by the speed of stirring.

**Figure 5:**
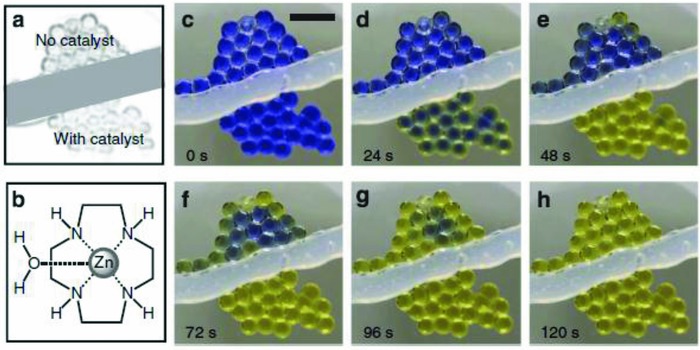
a–h) Optical images of silicone microcapsules containing a 3 wt% potassium carbonate solution core subjected to CO_2_ environment. Thymol blue was added to the liquid core to enable qualitative monitoring of capsule loading and unloading. Upon exposure to pure CO_2_, the MECS exhibited colorimetric change in pH (high to low), indicating the permeation of CO_2_ past the shell and absorption into the liquid core. The microcapsules at the bottom of each panel contain cyclen, a catalyst added to spur the absorption of CO_2_. Scale bar, 1 mm. Reproduced with permission.^94^ Copyright 2015, Springer Nature.

Microencapsulated sorbents as a new form of capture materials are considered to have the desirable characteristic features of both a solid and liquid sorbents. Aines et al. fabricated microencapsulated CO_2_ solvent (MECS), which are microscopic double emulsions of a thin layer of shell encapsulating a liquid solvent core.^93^, ^94^ The shell was made of hydrophobic photo‐polymerizable silicone rubber that allows the permeation of CO_2_ into the liquid core containing carbonate solutions. Carbonate solutions are being investigated as alternatives for CO_2_ absorbent, as they are abundant, environmentally benign, and resistant to degradation and have low volatility. The investigators successfully generated monodispersed MECS of 185 and 600 µm with wall thickness ranging from 10 to 50 µm in a microfluidic double capillary device. The MECSs were subjected to multiple absorption–desorption cycles under realistic thermal cycling conditions; with repeated loading and unloading of CO_2_ at varying temperatures ranging from 40 to 100 °C, yet the MECS retained their absorption capacity/performance within 90% of the initial equilibrium capacity without signs of rupturing. The regeneration of the MECS sorbents yielded purified CO_2_ that can be easily recovered. Cyclen (Zn‐1,4,7,10‐tetra‐azacyclododecane) was also added into the capsule cores to enhance the capture kinetics of the MECS. A colorimetric approach used to confirm the absorption of CO_2_ into the capsule core by addition of thymol blue, a pH indicator, is shown in **Figure** **5**.

Encapsulation allows the containment of solvents that are highly viscous, corrosive, or difficult to handle. In the case of encapsulated carbonate solutions, solid precipitation that forms following the reaction between CO_2_ and carbonate solution is easier to handle. Following the group's work on developing MECS, a more comprehensive study has been carried out to identify more polymeric materials with good permeability to CO_2_.^95^ Stolaroff et al. also developed two custom formulations of shell material, a silicone and an acrylate, which are promising for encapsulating water‐lean solvents. New classes of solvents such as ionic liquids (ILs), CO_2_‐binding organic liquids (CO_2_ BOLs) had also been examined. The advantages of ionic liquid over monoethanolamine (MEA) are the negligible vapor pressure which minimizes loss of ionic liquid and lower energy consumption for regeneration; the disadvantage however is the high viscosity which is mitigated by encapsulation.^96^ Capsules of Koechanol/water in thiol–ene, P_2222_benzimidazole/water in thiol–ene, and NDIL0231/water in SiTRIS had been successfully generated, which opens up more opportunities and research potentials for the encapsulation of ILs and CO_2_ BOLs. A list of functional microparticles and microspheres synthesized via different approaches for environmental treatment is included in **Table** **2**.

**Table 2:** Microparticles/microspheres for environmental remediation. While some are fabricated via microfluidic emulsification, others are synthesized via mechanical stirring or through suspension polymerization

Microparticles	Emulsion formation approach/solidification mechanism	Function	Size	Ref.
Polyethylenimine‐chitosan (PEI‐CS) microspheres	Microfluidic flow‐focusing chip; cross‐linking in solidification bath	Adsorption of copper ions in wastewater	378 µm (coefficient of variation (CV) = 2.3%)	97
Chitosan microparticles	Microfluidic flow‐focusing chip on PMMA plate; solidified under Schiff's base reaction	Methyl orange adsorption–dye treatment	735–1002 µm (CV = 1.86%)	98
Thiourea‐modified chitosan	PMMA plate microdevice with grafting; cross‐linking in solidification bath	Heavy metal wastewater–copper(II) ion removal	400–1500 µm	82
Poly(ionic liquid) microgel beads	Transparent microfluidic capillary reactor; UV photopolymerization	Heavy metal removal from wastewater, chromium(VI)	200–1000 µm	99
Chitosan/silica hybrid microspheres	PMMA plate microdevice; solidified under Schiff's base reaction	Copper(II) adsorption from wastewater	420 µm	84
Hollow silica microspheres	Cross‐flow microdevice; interfacial polymerization due to hydrolyzation and condensation	Waste removal and detoxification	91–137 µm	100
Microencapsulated sorbents (MECS)	Glass capillary microdevice; UV photopolymerization	Carbon capture	100–600 µm	94, 95
Graphene oxide microspheres	PDMS device with cross‐junction, UV photopolymerization	Removal of perfluorooctane sulfonate from polluted water	Not available	101
Carbon nanotube supraparticles (CNTSPs)	Flow‐focusing PDMS droplet generator	Removal of sodium dodecyl sulfate (SDS)	97.2 ± 10.7 µm	88
Magnetic microspheres with sodium alginate and activated carbon	Stirring and sonication	Removal of methylene blue	Not available	102
Chitosan microspheres	Mixing of chitosan and sodium triphosphate solution in ultrasound bath	Removal of oil from oil industry wastewater	500 µm	81
Spherical mesoporous polymer particles containing tunable amine	Suspension polymerization	Carbon dioxide capture	73–171 µm	92
Alginate microspheres containing biochar	Mechanical stirring	Immobilized phosphate ions	Not available	103

## 4 Other Microfluidics‐Inspired Technologies/Innovations

There have been many other innovations inspired by microfluidics for environmental application, such as graphene‐based microbots for the removal or recovery of toxic heavy metal from waste water streams.^104^ Graphene oxide and platinum nanolayers were sequentially electrochemically deposited onto the inner wall of the tubular micromotors or microbot, of average diameter of 4.6 ± 0.1 µm. Lead, Pb, was adsorbed onto the graphene layer, while the platinum layer decomposed hydrogen peroxide, H_2_O_2_, into water and oxygen forming microbubbles that propelled the movement of microbots, thereby improving the removal efficiency as microbots swim in the pollutant‐containing fluid. Layers of platinum and nickel were added to the microbots such that the motion of microbots could be controlled when magnetic force was applied externally. The microbots successfully removed 80% of Pb from the contaminated water having initial concentration of 1 ppm of Pb. Recovery of the microbots was possible by removing Pb in acidic solution. The regenerated microbots did not show sign of deterioration in their performances, while H_2_O_2_ could be added to the microbots for self‐propelling purpose. Scaling up seems possible for microbots, and this application is suitable for dye removal as well.

Indoor air quality monitoring is also now possible with a portable microdevice that carries out sampling and separation of fine particulate matter (PM).^105^ The device is useful for the monitoring of PM 2.5, known to be extremely hazardous to human respiratory system. Other applications include the study of marine zooplankton to changing environmental conditions and the ecological preferendum by loading plankton larvae into a microfluidic device.^106^ Yu et al. also reported the use of a microfluidic‐based mini‐metagenomics for identifying novel microbial lineages from complex environmental samples.^107^ Samples collected from two hot springs were partitioned and parallelized with microfluidic approach to generate multiple subsamples and to extract novel microbial genomes.

## 5 Conclusions and Future Outlooks

The demand for more efficient technologies to recover the ecosystems has seemingly brought about growing interest in research and development of microfluidic technologies for environmental applications. While conventional use of microfluidics has been on detection and analysis, droplet‐based microfluidics has recently diverted attention to the development of functional microparticles for environmental remediation. At present, however, the greatest challenge for the application of microfluidic systems in effective treatment of water and air pollutants remains to be both technical and economic barriers to industrialize microsystems or microtechnologies such that they can be employed or retrofitted into currently available technologies at industrial scale. The majority of the microsystems as they stand now are generally limited to “one‐design‐one‐application” basis, whereby due to highly functionalized design for each system, more customization work needs to be carried out to expand the functionality for other applications. Irrefutably, microfluidic technology is still at its infant stage and is an emerging area of technological development with great potential, especially in the area of environmental care.

Massive research work is still pouring into venturing varying microfluidic designs and applications. Efforts to address the challenges for on‐chip detection, such as poor sensitivity and selectivity, have led to slow but firm progress, thanks to the recent and ongoing research and development activities in these areas, as partly highlighted by the successful development of the coupling methodologies of the microdevices to analytical facilities that have effectively facilitated the automation of the integrated analytical systems.^108^, ^109^ The innovative use of microfluidic systems to produce solid sorbents has unlocked another possibility for microfluidic studies, and this calls for more vigorous studies to optimize the preparation and improve their performances for advanced applications. Material studies and development will be an integral part for the continuous advancement of microfluidic technologies. It remains debatable, however, as to whether or to what extent the microfluidic technologies will be able to effectively address the environmental challenges facing humanitarian, although no single technology can take on the role of tackling the whole environmental issues without the support of other technologies.

## Conflict of Interest

The authors declare no conflict of interest.
